# Alcohol withdrawal in patients with liver disease

**DOI:** 10.3389/fpsyt.2025.1569499

**Published:** 2025-05-26

**Authors:** Vugar Isazade, Asmaa Yehia, Umesh M. Sharma, Nan Zhang, Terry Schneekloth, Osama A. Abulseoud

**Affiliations:** ^1^ Department of Psychiatry and Psychology, Mayo Clinic Arizona, Phoenix, AZ, United States; ^2^ Department of Gastroenterology, Azerbaijan Medical University, Baku, Azerbaijan; ^3^ Department of Neuroscience, Graduate School of Biomedical Sciences, Mayo Clinic College of Medicine, Phoenix, AZ, United States; ^4^ Department of Medical Physiology, Faculty of Medicine, Mansoura University, Mansoura, Egypt; ^5^ Department of Hospital Internal Medicine, Mayo Clinic, Phoenix, AZ, United States; ^6^ Kern Center for the Science of Health Care Delivery, Mayo Clinic, Rochester, MN, United States; ^7^ Department of Psychiatry and Psychology, Mayo Clinic, Rochester, MN, United States

**Keywords:** alcohol withdrawal, sex difference, liver disease, CIWA-Ar, alcohol use disorder

## Abstract

**Objective:**

This study investigated and compared the clinical characteristics and treatment outcomes of alcohol withdrawal syndrome (AWS) in patients with and without liver diseases.

**Method:**

We conducted a retrospective chart review of all hospital admissions that received the CIWA-Ar protocol at the Mayo Clinic Health System between June 2019 and June 2022.

**Results:**

In this retrospective cohort study, we analyzed data for 1,586 hospitalizations for 811 liver disease [LIV(+)] patients and compared the results with 14,604 hospitalizations for 9,281 patients without liver disease [LIV(-)].Compared to the LIV(-) group, LIV(+) patients had more alcohol use disorder (94.3% vs. 58%, P = 0.003), longer hospital length of stay [Median (25^th^, 75^th^ percentiles): 93 (51,173) vs. 69 (43,125) hours, P = 0.001], longer time to reach peak CIWA-Ar scores [Mean ± SD: 26.3 ± 35.9 vs. 2.4 ± 32.5 hours, P = 0.001], lower first 24 hours lorazepam dose equivalents [3.5 (1.5, 7) vs. 3.5 (1.5, 8) mg, P = 0.001], and higher mortality rates (16.8% vs. 5.8%, P = 0.001). Within the LIV (+) cohort, no sex difference was depicted except for longer time to reach peak CIWA in males (Mean ± SD: 28.5 ± 40.3 vs. 21.7 ± 24.5 hours, P = 0.014).

**Conclusions:**

Our study highlights the higher mortality, hospital LOS, and ICU admissions in patients with liver cirrhosis and hepatic failure. We also recommend further controlled studies to examine the severity of AWS in hepatic patients, using other tools besides CIWA-Ar.

## 1 Introduction

Prolonged heavy alcohol consumption is known to cause deleterious effects on various organs (1), specifically the liver (2–4). Alcohol causes alcoholic fatty liver and alcoholic steatohepatitis, which could present as acute alcoholic hepatitis or as progressive hepatic fibrosis or cirrhosis (5, 6). Alcohol liver disease patients remain at high risk for morbidity due to liver failure, encephalopathy, bleeding varices, or hepatocellular carcinoma, and suffer from high mortality rates (7–10). The recent increase in alcohol sales (11), and per capita alcohol consumption (12, 13), specifically among women (14–18) resulted in a significant increase in liver diseases.

Complete abstinence is required to attenuate the progression of liver pathology and improve overall survival (19). However, many patients with liver disease continue to drink (20) or relapse within a short interval (19, 21). Alcohol withdrawal is a major contributor to relapse (22) because of persistent glutamatergic dysregulation (23) and increased post-withdrawal craving (24).

Alcohol withdrawal syndrome (AWS) is a common, life-threatening medical condition with a wide array of manifestations ranging from mild nausea and vomiting to anxiety, irritability, autonomic hyperactivity, seizures, and delirium tremens, which could be fatal (25, 26). The revised clinical institute withdrawal assessment for alcohol scale (CIWA-Ar) (27) is a 10-item scale [nausea and vomiting, tremors, paroxysmal sweats, anxiety, headache, agitation, tactile, auditory, and visual disturbances, and disorientation] commonly utilized to quantify the severity of AWS and administer lorazepam based on the severity score. Patients get placed on CIWA-Ar protocol for AWS when they report cessation of or drastic reduction in heavy alcohol intake, regardless of the presence or absence of AUD diagnosis. As many as 5.8% of all Veterans Affairs hospitalizations (n=469,082) in 2013 had AWS (28). Liver cirrhosis is a known risk factor for AWS (29), with about a third of patients hospitalized with alcohol-associated hepatitis having AWS (30).

Patients with liver diseases who continue to drink experience AWS when they get admitted to the hospital for decompensated liver conditions or for other medical disorders (31). In patients with liver cirrhosis, distinguishing AWS from hepatic encephalopathy (HE) is particularly challenging. Both conditions can present with altered mental status, but their management differs significantly. HE often requires treatments like lactulose, whereas AWS is typically managed with benzodiazepines. However, in severe liver disease, benzodiazepines can precipitate or worsen HE, necessitating careful selection and dosing of these medications (32). Considering this complexity, it’s crucial to investigate the difference in AWS manifestations, hospital course and treatment outcomes between hepatic and non-hepatic patients to better characterize AWS in patients with liver diseases. In this study, we hypothesized that patients with liver disease will have more severe withdrawal, require more benzodiazepines, and suffer higher mortality rates. We also examined the differences between male and female hepatic patients.

## 2 Methods

The study was approved by the Institutional Review Board of Mayo Clinic (ID#22-008591) in compliance with all international and institutional research standards. We retrieved the electronic medical records of all hospital admissions who were placed under the CIWA-Ar protocol for alcohol withdrawal at Mayo Clinic Health System from June 2019 through June 2022. We only included patients ≥21 years old and previously published a study examining patients under 21 years old for AWS (33). The CIWA-Ar scale is a 10-item survey used to quantify the severity of withdrawal manifestations (27). Senior data analysts initially obtained all patient data from an electronic data extraction. Active problem lists were used to identify medical and psychiatric comorbidities. Patients who had liver cirrhosis and liver failure were considered under liver-positive (LIV+) group.

The first admission for each patient was included in the analysis for demographics and comorbid medical and psychiatric conditions in cases of multiple encounters for a single patient. Data for all hospitalization episodes included admission laboratory values, each CIWA-Ar assessment from admission until discharge, hospital course including intensive care unit (ICU) admissions, benzodiazepine treatment, and all-cause mortality. Administered benzodiazepine doses were converted to lorazepam-equivalent doses (34). Mortality data were ascertained through June 2023.

### 2.1 Statistical analysis

The normality of continuous variables was assessed using the Kolmogorov-Smirnov test. Data were presented as mean ± standard deviation (SD) if normally distributed or as a median, interquartile range (IQR), and range if not normally distributed. Categorical data were expressed as percentages. To compare continuous variables between patients with and without comorbid liver disease or between males and females within the liver disease group, we utilized the student’s t-test for normally distributed data and the Mann-Whitney U test for non-parametric data. Categorical variables were compared using the chi-square test or Fisher’s exact test. Adjustment for multiple comparisons was implemented using Bonferroni correction to reduce the risk of false positive results. P values ≤ 0.05 were considered statistically significant. All analyses were performed using BlueSky Statistics version 10.3.1, standard SPSS software version 28.0 (IBM Corp.), and PRISM GraphPad 10.1.2 (La Jolla, CA).

## 3 Results

### 3.1 Liver diseases

Between June 2019 and June 2022, we identified a total number of 10,092 patients with 16,190 hospital admissions based on the implementation of the CIWA-Ar protocol for AWS. Within this cohort, 811 (8%) patients had liver diseases [LIV(+)] with 1586 (9.8%) hospital admissions. The LIV(+) group included 559 (68.9%) males and 252 females (31.1%).

### 3.2 Demographics

The mean age of the AWS LIV(+) group was significantly higher than the LIV(-) group [55.3 ± 12.6 vs. 52.3 ± 16.1, P=0.002], with male LIV(+) patients being significantly older than their female counterparts (56.3 ± 12.5 vs. 53.2 ± 12.7, P=0.025). Compared to the LIV(-) group, LIV(+) patients included less African Americans (1.6% vs. 4.2%, P = 0.002). LIV(+) patients were more likely to be unemployed (64.6% vs. 52.2%, P = 0.002), with a higher BMI [28.7 ± 7.2 vs. 27.8± 6.6kg/m^2^, P =0.005], except for the 18.5-24.9 BMI category (LIV (+) vs. LIV (-): 27.4% vs. 32.9%, P=0.025]. Within the LIV (+) cohort, males had a higher BMI [29.5 ± 7.1 vs. 26.9 ± 7.1 kg/m^2^, P < 0.002] (**Table 1**).

**Table 1:** Demographics.

Demographics		Liv (+) patients (n=811)	Liv (-) patients (n=9,281)	P-Value	P_corr_-Value	Liv (+) Males (n=559)	Liv (+) Females (n=252)	P-Value	P_corr_-Value
Age years Mean ± SD, (Min-Max)		55.3 ± 12.6 (23-88)	52.3 ± 1’6.1 (21-100)	<0.0001	0.002	56.3 ± 12.5 (23-87)	53.2 ± 12.7 (25-88)	0.001	0.025
Race	White	726 (89.5%)	8319 (89.6%)	0.9	ns	505 (90.3%)	221 (88%)	0.3	ns
	African-American	13 (1.6%)	393 (4.2%)	0.0001	0.002	10 (1.8%)	3 (1.2%)	0.7	ns
	Other	55 (6.8%)	394 (4.2%)	0.001	0.025	6.3%	20 (8%)	0.3	ns
	Unknown	17 (2.1%)	175 (1.9%)	0.6	ns	9 (1.6%)	8 (3.2%)	0.18	ns
Ethnicity	Non-Hispanic	757 (93.3%)	8704 (93.8%)	0.5	ns	519 (92.8%)	238 (94.8%)	0.3	ns
	Hispanic	32 (3.9%)	296 (3.2%)	0.2	ns	25 (4.5%)	7 (2.8%)	0.3	ns
	Unknown	22 (2.7%)	281 (3.0%)	0.7	ns	15 (2.6%)	7 (2.8%)	0.99	ns
Employment Status	Unemployed	524 (64.6%)	4845 (52.2%)	<0.0001	0.002	354 (63.3%)	170 (67.7%)	0.2	ns
	Employed	216 (26.6%)	3542 (38.2%)	<0.0001	0.002	153 (27.4%)	63 (25.1%)	0.5	ns
	Student	0 (0.0%)	39 (0.4%)	0.07	ns	0 (0%)	0 (0.0%)	0.99	ns
	Unknown	71 (8.8%)	855 (9.2%)	0.7	ns	52 (9.3%)	19 (7.6%)	0.5	ns
Marital Status	Single	309 (38.1%)	3686 (39.7%)	0.3	ns	229 (40.9%)	80 (31.9%)	0.015	ns
	Married	258 (31.8%)	3238 (34.9%)	0.08	ns	168 (30.1%)	90 (35.9%)	0.1	ns
	Divorced	170 (21%)	1506 (16.2%)	0.0007	0.017	118 (21.1%)	52 (20.7%)	0.9	ns
	Widowed	37 (4.6%)	502 (5.4%)	0.3	ns	21 (3.8%)	16 (6.4%)	0.1	ns
	Others	25 (3.1%)	254 (2.7%)	0.5	ns	16 (2.9%)	9 (3.6%)	0.6	ns
	Unknown	12 (1.5%)	90 (1.0%)	0.19	ns	7 (1.3%)	5 (2.0%)	0.5	ns
BMI (kg/m^2^) Mean ± SD, (Min-Max)		28.7 ± 7.2 (13.8-62.9)	27.8 ± 6.6 (11.9-71.9)	0.0002	0.005	29.5 ± 7.1 (13.8-62.9)	26.9 ± 7.1 (14.0-58.9)	<0.0001	0.002
BMI (kg/m^2^) categories	BMI <18.5	26 (3.2%)	267 (2.9%)	0.5	ns	14 (2.5%)	12 (1.5%)	0.1	ns
	BMI 18.5-24.9	222 (27.4%)	3057 (32.9%)	0.001	0.025	128 (22.9%)	94 (11.6%)	<0.0001	0.002
	BMI 25-29.9	263 (32.4%)	2756 (29.7%)	0.1	ns	186 (33.3%)	77 (9.5%)	0.5	ns
	BMI 30-39.9	229 (28.2%)	2248 (24.2%)	0.012	ns	178 (31.8%)	51 (6.3%)	0.0007	0.017
	BMI≥40	59 (7.3%)	438 (4.7%)	0.002	0.05	47 (8.4%)	12 (1.5%)	0.07	ns
	BMI unknown	12 (1.5%)	515 (5.5%)	<0.0001	0.002	6 (1.1%)	6 (0.7%)	0.2	ns

### 3.3 Comorbid medical and psychiatric conditions

Compared to LIV (-) patients, LIV (+) group had a significantly higher prevalence of alcohol use disorder (AUD: 94.3% vs. 58%, P = 0.003), gastrointestinal (84.1% vs. 36.9%, P = 0.003), Neurological (42.2% vs. 31%, P = 0.003), respiratory (29.5% vs. 21.5%, P = 0.003), Renal (24.8% vs. 14.6%, P = 0.003), nutritional (21.3% vs. 8.9%, P =0.003), dermatological (13.2% vs. 8.5%, P = 0.003), hematological disorders (6.8% vs. 2.1%, P = 0.003) and infections (25.8% vs. 12.3%, P = 0.003), but a lower prevalence of bipolar disorder (2.3% vs. 5%, P = 0.003), suicidal ideations (1% vs. 4.9%, P = 0.003), and ADHD (0.2% vs. 2%, P=0.003). Within the LIV (+) group males had significantly more cardiovascular disorders (58.7% vs. 42.9%, P = 0.003), while females had more depression (38.9% vs. 26.5%, P = 0.018) (**Table 2**).

**Table 2:** Comorbid medical and psychiatric conditions.

Disorders	Liv (+) patients (n=811)	Liv (-) patients (n=9,281)	P-Value	P_corr_-Value	Liv (+) Males (n=559)	Liv (+) Females (n=252)	P-Value	P_corr_-Value
Alcohol use disorder	765 (94.3%)	5383 (58.0%)	<0.0001	0.003	533 (95.3%)	232 (92.1%)	0.07	ns
Gastrointestinal	682 (84.1%)	3429 (36.9%)	<0.0001	0.003	469 (83.9%)	213 (84.5%)	0.9	ns
Elevated liver transaminases	44 (5.4%)	243 (2.6%)	<0.0001	0.003	31 (5.5%)	13 (5.2%)	0.8	ns
Liver Transplant	13 (1.6%)	28 (0.3%)	<0.0001	0.003	8 (1.4%)	5 (2.0%)	0.5	ns
Viral Hepatitis	111 (13.7%)	127 (1.4%)	<0.0001	0.003	79 (14.1%)	32 (12.7%)	0.6	ns
Cardiovascular	436 (53.8%)	4518 (48.7%)	0.006	ns	328 (58.7%)	108 (42.9%)	<0.0001	0.003
Anemia	355 (43.8%)	1343 (14.5%)	<0.0001	0.003	233 (41.7%)	122 (48.4%)	0.07	ns
Neurological	342 (42.2%)	2877 (31.0%)	<0.0001	0.003	230 (41.1%)	112 (44.4%)	0.3	ns
Endocrinological	321 (39.6%)	3257 (35.1%)	0.011	ns	220 (39.4%)	101 (40.1%)	0.8	ns
Depression	246 (30.3%)	2805 (30.2%)	0.9	ns	148 (26.5%)	98 (38.9%)	0.0005	0.018
Generalized anxiety	188 (23.2%)	2293 (24.7%)	0.3	ns	116 (20.8%)	72 (28.6%)	0.019	ns
Respiratory	239 (29.5%)	1998 (21.5%)	<0.0001	0.003	170 (30.4%)	69 (27.4%)	0.4	ns
Renal	201 (24.8%)	1352 (14.6%)	<0.0001	0.003	145 (25.9%)	56 (22.2%)	0.2	ns
Infections	209 (25.8%)	1141 (12.3%)	<0.0001	0.003	143 (25.6%)	66 (26.2%)	0.8	ns
Orthopedic	155 (19.1%)	1414 (15.2%)	0.004	ns	105 (18.8%)	50 (19.8%)	0.7	ns
Nutritional	173 (21.3%)	824 (8.9%)	<0.0001	0.003	114 (20.4%)	59 (23.4%)	0.3	ns
History of childhood abuse	119 (14.7%)	1457 (15.7%)	0.4	ns	81 (14.5%)	38 (15.1%)	0.8	ns
History of traumatic injuries	112 (13.8%)	1255 (13.5%)	0.8	ns	76 (13.6%)	36 (14.3%)	0.8	ns
Dermatological	107 (13.2%)	788 (8.5%)	<0.0001	0.003	77 (13.8%)	30 (11.9%)	0.5	ns
Ear Nose and Throat (ENT)	80 (9.9%)	680 (7.3%)	0.012	ns	52 (9.3%)	28 (11.1%)	0.4	ns
Substance dependence	63 (7.8%)	827 (8.9%)	0.3	ns	46 (8.2%)	17 (6.7%)	0.5	ns
Sleep disorders	68 (8.4%)	678 (7.3%)	0.2	ns	46 (8.2%)	22 (8.7%)	0.7	ns
Malignancy	75 (9.2%)	807 (8.7%)	0.6	ns	53 (9.5%)	22 (8.7%)	0.7	ns
Ophthalmological	49 (6.0%)	462 (5.0%)	0.18	ns	36 (6.4%)	13 (5.2%)	0.5	ns
Hematologic	55 (6.8%)	197 (2.1%)	<0.0001	0.003	35 (6.3%)	20 (7.9%)	0.3	ns
Altered mental status	42 (5.2%)	373 (4.0%)	0.11	ns	26 (4.7%)	16 (6.3%)	0.3	ns
Post-traumatic stress disorder (PTSD)	23 (2.8%)	434 (4.7%)	0.013	ns	18 (3.2%)	5 (2.0%)	0.3	ns
Bipolar	19 (2.3%)	463 (5.0%)	0.0003	0.01	11 (2.0%)	8 (3.2%)	0.3	ns
History of suicidal ideations	8 (1.0%)	451 (4.9%)	<0.0001	0.003	6 (1.1%)	2 (0.8%)	0.99	ns
Adjustment disorder	8 (1.0%)	161 (1.7%)	0.11	ns	3 (0.5%)	5 (2.0%)	0.11	ns
History of suicide attempts	6 (0.7%)	188 (2.0%)	0.007	ns	3 (0.5%)	3 (1.2%)	0.3	ns
Attention deficit hyperactivity disorder (ADHD)	2 (0.2%)	185 (2.0%)	<0.0001	0.003	2 (0.4%)	0 (0.0%)	0.99	ns
Schizoaffective	4 (0.5%)	81 (0.9%)	0.3	ns	2 (0.4%)	2 (0.8%)	0.5	ns
Eating	2 (0.2%)	35 (0.4%)	0.7	ns	0 (0.0%)	2 (0.8%)	0.09	ns
Conversion	0 (0.0%)	29 (0.3%)	0.16	ns	0 (0.0%)	0 (0.0%)	0.99	ns
Schizophrenia	1 (0.1%)	79 (0.9%)	0.02	ns	1 (0.2%)	0 (0.0%)	0.99	ns

### 3.4 Laboratory results

LIV (+) and LIV(-) patients had no significant difference in BAC. LIV(+) patients exhibited higher mean alkaline phosphatase [ALP: 179.5 ± 180.3 vs. 109.1 ± 85.62 IU/L, P = 0.001], aspartate aminotransferase [AST: 129.4 ± 147.4 vs. 99.26 ± 260.9 IU/L, P = 0.026] and blood urea nitrogen [BUN: 17.79 ± 18.33 vs. 15.28 ± 13.4 mg/dL, P = 0.001]. However, they had a lower prevalence of positive cannabis (THC) results in urine drug screen (5.9% vs. 9.4%, P=0.001).Within the LIV (+) group, more females had BAC level of 201–400 mg/dL (13.8% vs. 8%. P= 0.013), with no other differences in lab results. (**Table 3**).

**Table 3:** Laboratory values at time of admission.

Laboratory values	Liv (+) (n=811 patients & 1,586 hospitalizations)	Liv (-) (n=9,281 patients & 14,604 hospitalizations)	P-Value	P_corr_-Value	Liv (+) Males (n=559 patients & 1,075 hospitalizations)	Liv (+) Females (n=252 patients & 511 hospitalizations)	P-Value	P_corr_-Value
Tested for blood alcohol [n(%)]	360 (22.9%)	3,520 (24.1%)	0.3	ns	216 (20.3%)	144 (28.3%)	0.0005	0.006
BAC mg/dL [(Mean ± SD), Min-Max, (n)]	[(202.1 ± 140.7) 10-544 (n=360)]	[(217.2 ± 136.1) 10-679.2 (n=3,520)]	0.046	ns	[(201 ± 142) 10-536 (n=216)]	[(203.9 ± 139.1) 10-544 (n=144)]	0.8	ns
BAC <80 mg/dL [n(%)]	103 (6.6%)	760 (5.2%)	0.025	ns	62 (5.8%)	41 (8.1%)	0.1	ns
BAC 81–200 mg/dL [n(%)]	77 (4.9%)	805 (5.5%)	0.3	ns	49 (4.6%)	28 (5.5%)	0.4	ns
BAC 201–400 mg/dL [n(%)]	152 (9.7%)	1,640 (11.2%)	0.06	ns	85 (8.0%)	67 (13.2%)	0.001	0.013
BAC >400 mg/dL [n(%)]	28 (1.8%)	315 (2.2%)	0.3	ns	20 (1.9%)	8 (1.6%)	0.8	ns
THC [n positive (%)]	93 (5.9%)	1,367 (9.4%)	<0.0001	0.001	70 (6.6%)	23 (4.5%)	0.11	ns
BUN (mg/dL) [(Mean ± SD), Min-Max, (n)]	[(17.79 ± 18.33) 1-99 (n=845)]	[(15.28 ± 13.4) 1-122 (n=6,731)]	<0.0001	0.001	[(19.03 ± 18.86) 1-99 (n=552)]	[(15.44 ± 17.1) 1-97.4 (n=293)]	0.006	ns
Creatinine (mg/dL) [(Mean ± SD), Min-Max, (n)]	[(2.24 ± 15.59) 0.29-449 (n=1,512)]	[(2.04 ± 87.16) 0.29-10,165 (n=1,3654)]	0.9	ns	[(2.26 ± 12.57) 0.34-296 (n=1,019)]	[(2.18 ± 20.49) 0.29-449 (n=493)]	0.9	ns
ALP (IU/L) [(Mean ± SD), Min-Max, (n)]	[(179.5 ± 180.3) 29-3,835 (n=799)]	[(109.1 ± 85.62) 15-992 (n=5,637)]	<0.0001	0.001	[(172.5 ± 136) 49-1,315 (n=516)]	[(192.3 ± 240.7) 29-3,835 (n=283)]	0.13	ns
ALT (IU/L) [(Mean ± SD), Min-Max, (n)]	[(91.98 ± 475.5) 6-9,700 (n=813)]	[(72.16 ± 211.1) 4-9,395 (n=5,791)]	0.04	ns	[(92.52 ± 410.8) 6-6,026 (n=527)]	[(90.98 ± 576.9) 8-9,700 (n=286)]	0.9	ns
AST (IU/L) [(Mean ± SD), Min-Max, (n)]	[(129.4 ± 147.4) 10-999 (n=750)]	[(99.26 ± 260.9) 7-9,440 (n=5,362)]	0.002	0.026	[(126.5 ± 146.5) 10-999 (n=493)]	[(134.8 ± 149.1) 14-963 (n=257)]	0.4	ns
TSH (mIU/L) [(Mean ± SD), Min-Max, (n)]	[(3.6 ± 6.354) 0.04-98.3 (n=397)]	[(2.99 ± 6.38) 0.005-136.3 (n=4,741)]	0.06	ns	[(3.20 ± 4.23) 0.1-42.9 (n=243)]	[(4.23 ± 8.69) 0.04-98.3 (n=154)]	0.11	ns

### 3.5 Hospital course

The mean hospital length of stay (LOS) was significantly longer in LIV (+) patients [LIV (+) vs. LIV (-): 93 (51,173) vs. 69 (34,125) hours, P = 0.001]. LIV (+) patients were more likely to be admitted to the ICU (29.3% vs. 23%, P=0.001), with no difference in ICU LOS and with no sex difference.

There was no significant difference in the median peak total CIWA-Ar score between LIV(+) and LIV(-) patients or within the LIV(+) group. However, the time to reach peak withdrawal severity was lower in LIV (+) compared to LIV (-) patients (14 (5,33) vs. 11(4,28) hours, P = 0.001). Within the LIV(+) group, males had a significantly higher time to reach peak withdrawal severity than females (14 (5,34) vs. 14 (5,30), P = 0.014).

The percentage of patients who received benzodiazepine treatment over the whole hospital LOS and during the first 24 hours of admissions and the total LOS lorazepam dose equivalent showed no difference between LIV(+) and LIV(-), nor between males and females LIV(+) patients. However, during the first 24 hours of admissions, LIV (+) patients had lower lorazepam dose equivalent than LIV(-) ones (3.5 (2,15) vs. 5 (2,14.6) mg, P = 0.001), with no sex difference within the LIV(+) group.

We collected all-cause mortality data during hospitalization and after discharge through June 2023. Sixteen percent of patients (n=266) in the LIV (+) group died, which was significantly higher than patients without liver diseases [LIV (+) vs LIV (-): 16.8% vs. 5.8%, P = 0.001]. There was no significant difference between males and females in the LIV (+) group. In the LIV (+) patients, 2.7% (n=42) died during their hospitalization, which was significantly higher than deaths in the non-liver group (0.8%, n=116, P = 0.001). No significant difference was detected between sex groups in the in-hospital mortality in patients with liver diseases. On the other hand, 14.1% of LIV (+) patients died during the post-hospitalization period which was significantly higher than the post-hospitalization mortality in the LIV (-) group (5%, P = 0.001). No significant difference was observed between males and females in the LIV (+) group. Furthermore, the median time between hospital discharge and death was significantly shorter in the LIV (+) group [0.33 (0.06, 0.87) vs. 0.59 (0.2, 1.3) years, P = 0.011], with no sex difference. (**Table 4**).

**Table 4:** Hospital course.

Hospital course	Liv (+) (n=811 patients & 1,586 hospitalizations)	Liv (-) (n=9,281 patients & 14,604 hospitalizations)	P-Value	P_corr_-Value	Liv (+) Males (n=559 patients & 1,075 hospitalizations)	Liv (+) Females (n=252 patients & 511 hospitalizations)	P-Value	P_corr_-Value
Hospital LOS (hours) [median (25th, 75th percentiles), mean ± SD)]	93 (51, 173), 154.4 ± 239.5	69 (34, 125), 115.9 ± 210.7	<0.0001	0.001	95 (51, 173), 161.5 ± 269.4	92 (50, 168), 139.4 ± 158.3	0.08	ns
Required ICU admissions [n(%)]	464 (29.3%)	3364 (23.0%)	<0.0001	0.001	314 (29.2%)	150 (29.4%)	0.9	ns
ICU LOS (hours) [median (25th, 75th percentiles), mean ± SD)]	46.2 (24.3, 88.9), 68.1 ± 66.1	42.8 (23.4, 74.1), 63.7 ± 70.7	0.2	ns	45.8 (24.9, 91.3), 69.7 ± 71.3	47.5 (23.7, 83), 64.8 ± 53.9	0.4	ns
Patients with a Peak CIWA score ≥4 [n(%)]	1315 (82.9%)	11759 (80.5%)	0.022	ns	883 (82.1%)	432 (84.5%)	0.25	ns
Peak total CIWA-Ar Score [median (25th, 75th percentiles), mean ± SD)]	11 (7, 17), 13.1 ± 7.7	12 (7, 18), 13.6 ± 7.9	0.039	ns	12 (7, 18), 13.4 ± 7.9	11 (7, 16), 12.5 ± 7.1	0.031	ns
Time (hours) from admission to peak total CIWA-Ar Score [median (25th, 75th percentiles), mean ± SD)]	14 (5, 33), 26.3 ± 35.9	11 (4, 28), 22.4 ± 32.5	<0.0001	0.001	14 (5, 34), 28.5 ± 40.3	14 (5, 30), 21.7 ± 24.5	0.001	0.014
Patients received Benzodiazepien during the first 24 hours of hospitalization [n(%)]	849 (53.5%)	8097 (55.4%)	0.15	ns	571 (53.1%)	278 (54.4%)	0.6	ns
First 24 hr Lorazepam dose equivalent (mg) [median (25th, 75th percentiles), mean ± SD)]	3.5 (1.5, 7), 5.5 ± 6.0	3.5 (1.5, 8), 6.7 ± 8.5	<0.0001	0.001	4 (2, 7.5), 5.8 ± 6.4	3 (1.4, 7), 4.9 ± 5.2	0.042	ns
Patients received Benzodiazepien over the whole hospital LOS [n(%)]	1011 (63.8%)	9389 (64.3%)	0.6	ns	678 (63.1%)	333 (65.2%)	0.4	ns
Whole LOS Lorazepam dose equivalent (mg) [median (25th, 75th percentiles), mean ± SD)]	5.5 (2, 15), 16.1 ± 41.3	5 (2, 14.6), 16.1 ± 37.9	0.9	ns	6 (2, 17), 18.5 ± 48.7	5 (2, 12.8), 18.5 ± 48.7	0.007	ns
All-cause mortality [n(%)]	266 (16.8%)	846 (5.8%)	<0.0001	0.001	183 (17.0%)	83 (16.2%)	0.7	ns
In-hospital mortality [n(%)]	42 (2.7%)	116 (0.8%)	<0.0001	0.001	30 (2.8%)	12 (2.4%)	0.7	ns
Post-hospitalization mortality [n(%)]	224 (14.1%)	730 (5.0%)	<0.0001	0.001	153 (14.2%)	71 (13.9%)	0.8	ns
Duration (years) between hospitalization and death [median (25th, 75th percentiles), mean ± SD)]	0.33 (0.06, 0.87), 0.63 ± 0.73	0.59 (0.2, 1.3), 0.82 ± 0.76	0.0008	0.011	0.37 (0.07, 0.88), 0.64 ± 0.73	0.29 (0.05, 1.0), 0.59 ± 0.73	0.6	ns

## 4 Discussion

In this retrospective study, we examined potential differences in the clinical characteristics and treatment outcomes of AWS between patients with and without liver diseases and between males and females within the liver disease group. Despite the fact that 94.3% of LIV(+) and 58% of LIV(-) patients were recorded to have AUD diagnosis, being placed on CIWA-Ar indicates that all the patients were treated for AWS regardless the presence or absence of documented AUD diagnosis. However, AUD diagnosis documentation could have been missing due to limited history collection during acute illness and ICU settings. We hypothesized worse withdrawal manifestations and course in patients experiencing alcohol withdrawal with concurrent hepatic dysfunction compared to those without hepatic comorbidity. Intriguingly, our findings reveal that patients with hepatic involvement exhibited longer hospital stays, more frequent ICU admissions. They had a higher likelihood of mortality during and after hospitalization, and died within a shorter time interval, reflecting a need for closer observation of the course of AWS in hepatic patients. We also found delayed time to peak CIWA-Ar scores, and lower benzodiazepine usage in the first 24 hours of withdrawal management in hepatic patients and, to our surprise, no differences were depicted in the peak CIWA scores or in the total benzodiazepine dose during the whole hospital LOS.

In addition, examining sex differences within the LIV (+) group showed no difference between males and females, except for a longer time to reach peak CIWA in males. Furthermore, our study provides a detailed description of the prevalence of individual medical and psychiatric comorbidities in a large cohort of patients admitted to the hospital for different reasons and treated with the CIWA-Ar protocol for AWS.

AWS develops more frequently in actively drinking patients who require hospital admission for decompensation or other-related comorbidity due to the abrupt cessation of alcohol use. Therefore, a close observation of those patients is most needed (31). The incidence of AWS in hospitalized patients is estimated to be between 1% and 5% (35, 36). Hepatologist-treated inpatients have unquestionably higher rates of AWS; in one nationwide sample of patients at Veterans Administration hospitals, 14% of patients admitted for liver injury developed AWS, and cirrhosis was linked to a higher risk of developing AWS during inpatient stays (29).

In our study, the finding of no difference in peak CIWA score and total benzodiazepine dose opposes our hypothesis. We expected withdrawal manifestations and treatment outcomes to be different in patients with liver disease for several reasons. First, the reduced hepatic alcohol dehydrogenase (ADH) enzyme activity can result in higher BAC levels, which we didn’t observe in our data (37). Second, benzodiazepines, the gold standard for AWS treatment (38) are all metabolized in the liver (39), which might cause hepatic patients to necessitate higher doses, which we also didn’t observe in our data. Although there are reports of liver cirrhosis being a risk factor for development of AWS (29), a systematic review of 15 studies, showed that history of chronic liver disease is not a risk factor for AWS severity (40). Interestingly, Monte et al. reported that liver cirrhosis is a risk factor for mortality among those admitted for treatment of alcohol withdrawal, which is consistent with our finding of higher mortality rates among hepatic patients (41). The observed higher time to reach peak CIWA among hepatic patients shows a potential need for longer monitoring during the course of AWS in hepatic patients.

Despite the fact that we see no difference in the peak CIWA score between liver and non-liver patients, this does not necessarily indicate no difference in severity. CIWA-Ar, the tool used to measure AWS severity, is a 10-item scale of common signs and symptoms of alcohol withdrawal. CIWA-Ar can be confounding in the presence of a comorbid medical condition, especially in the differentiation between delirium tremens and medically related delirium (42). In addition, in a study that included all ICU-admitted adults after treatment for AWS, using CIWA-Ar to assess AWS severity and response to treatment, Steel et al. tested the association between patient characteristics and CIWA-Ar monitoring. They found that CIWA-Ar monitoring was used inconsistently in ICU patients with AWS and was completed less often in those who were intubated or identified as Black (43). Therefore, alternative methods for severity assessment are needed, especially since we found more frequent ICU admissions among our hepatic patients.

The prevalence of hazardous alcohol consumption and binge drinking has notably increased among women (14, 16, 44–46). Women were reported to be more susceptible than men to alcohol-induced hepatotoxicity and neurotoxicity (47). Women achieve higher BAC than men after ingesting the same dose of ethanol per kilogram of body weight, possibly because of the distribution of ethanol on a smaller body water content in women as compared with men (47). In addition, women have faster hepatic oxidation of ethanol, which accentuates alcohol toxicity by increasing the generation of acetaldehyde (48). Despite these reports, we didn’t detect differences in the AWS severity, benzodiazepine dose needed, or hospital course. The only observation in our data was longer time to reach peak CIWA in males compared to females, which still needs controlled studies to investigate the potential sex difference.

As many as 16.8% of AWS patients with liver diseases died either in the hospital or within a median of 0.3 years after hospitalization. This rate of death is more than double the rate in the LIV (-) group. The mortality rate in patients with AWS was estimated to be 6.6% in a previous study of 436 AWS patients (539 hospitalizations) (41). Another study from Spain showed significantly higher long-term mortality in individuals with AWS (n=1,265) compared to a reference cohort (n=1,362) of individuals from the same area [8.6% (95% CI: 7.7-9.7)] (49). Existing literature indicates that mortality rates among AWS patients are higher in males than in females (46); however, there was no significant difference in our findings. Additionally, the literature suggests that the time from initial hospitalization to death is typically shorter in males with AWS than in females (46); however, our study did not report a significant sex difference. Notably, we observed a shorter interval between hospitalization and mortality in LIV (+) patients than in LIV (-) patients. In our study, we do not have data on the cause of death for these patients. However, it is plausible to speculate that liver cirrhosis plays a role in this high mortality rate. Recent epidemiological data document a three-fold increase in the alcoholic cirrhosis mortality rate from 3.3 per 100,000 in 1999 to 10.6 per 100,000 in 2019 (50).

The results of this study should be viewed in the context of its strengths and limitations. Our large sample size, specifically females, detailed phenotyping for comorbid conditions, and hospital course all add more robustness to the results. On the other hand, the retrospective nature of the study, with no access to data on drinking patterns, the time from last drink to hospital admission, the extent and duration of liver disease, the racial homogeneity (predominantly white and non-Hispanic, and fewer African Americans among liver patients), and the lack of data on received medications other than benzodiazepines could limit the generalizability of the results. In addition, as many as 42% in the LIV + and 6% in the LIV- groups did not have an AUD diagnosis mentioned in their problem list, possibly because a detailed history of alcohol consumption was not collected by their treating physicians. Despite these limitations, our study highlights the higher mortality, hospital LOS, and ICU admissions in patients with liver cirrhosis and hepatic failure. We also recommend further controlled studies to examine the severity of AWS in hepatic patients, using other tools besides CIWA-Ar.

## Data Availability

The data analyzed in this study is subject to the following licenses/restrictions: Mayo Clinic regulates access to patient data. Requests to access these datasets should be directed OA, abulseoud.osama@mayo.edu.
